# Effects of statins on multispecies oral biofilm

**DOI:** 10.1080/20002297.2017.1325249

**Published:** 2017-05-30

**Authors:** Marta Kamińska, Ardita Aliko, Annelie Hellvard, Agata Marczyk, Piotr Mydel

**Affiliations:** ^a^ Broegelmann Research Laboratory, Department of Clinical Science, University of Bergen, Bergen, Norway

## Abstract

Statins effectively reduce the risk of cardiovascular-related morbidity and mortality in patients with hyperlipidemia, hypertension or type-II diabetes. In addition to lowering cholesterol, several studies have attributed statins with immunomodulatory and bactericidal properties. In this context, the aim of this study was to obtain information about their antimicrobial activity against key bacteria populating oral biofilms and relevant in periodontitis. Using the planktonic monocultures and multispecies biofilm models to assess the impact of the four statins here investigated, we demonstrated their high efficacy against *Porphyromonas gingivalis* (*P. gingivalis*) also leading to a significant decrease in cumulative bacterial load in early biofilm. Conversely, in established biofilm, simvastatin decreased *P. gingivalis* counts by up to more than 1ʹ000-fold, but, in contrast with early biofilm, *Streptococcus gordonii* expanded significantly to populate this emerging niche and compensate for diminishing *P. gingivalis* counts. These findings allow for speculations that similar events, when occurring *in vivo*, could initiate a shift of the oral microflora from a pathogenic to a more commensal state. Thus, we believe that simvastatin should be studied as an exemplary drug for periodontitis treatment.

